# Gas chromatography – Mass spectrometry (GC-MS) profiling reveals newly described bioactive compounds in *Citrullus colocynthis* (L.) seeds oil extracts

**DOI:** 10.1016/j.heliyon.2023.e16861

**Published:** 2023-06-03

**Authors:** Faten Hameed Thamer, Noah Thamer

**Affiliations:** Department of Chemistry, Sana’a University, Sana’a, Yemen

**Keywords:** Phytoconstituents, *Citrullus colocynthis* (L.), GC-MS, Pharmacology, Seeds oil, Carotenes, Isooctylphthalat

## Abstract

*Citrullus colocynthis* (L.) (*C. colocynthis)*, commonly known as Handal in Yemen and other Arabic countries, is a plant with a wide range of pharmacological properties. These properties are attributed to secondary metabolites, known as phytochemicals, present in the plant. In this study, the seed of *C. colocynthis* were extracted using dichloromethane, and the resulting oil extract was screened to identify active phytoconstituents using gas chromatography – Mass spectrometry (GC-MS). The gas chromatography – Mass spectrometry is used to identification of the phytoconstituents and the spectrum of unknown compounds were compared with the compounds stored in the National Institute of Standards and Technology Mass Spectral database (NIST) and WILEY library of GC-MS. A total of fifty five compounds appeared in GC-MS chromatogram, twenty-four major bioactive compounds were identified in the present study. The major components of the oil extract were found to be carotenes, phenols, esters, and steroids. From the GC-MS chromatogram of dichloromethane seeds oil extract, some of the identified components possess pharmacological actions as per information available in the literature. One of the major component identified in the *C. colocynthis* seed oil extract was isooctylphthalate (58%), which exhibit strong antimicrobials effect. Therefore, *C. colocynthis* is considered to be a natural source of isooctylphthalate. From the results, this study is the first to report the presence of various bioactive components of phytopharmaceutical importance in *C. colocynthis*.

## 1 Introduction

Medicinal plants are rich in secondary metabolites with many biological activities including antioxidant, anti-inflammatory, anticancer, antiviral, antifungal, and antibacterial agents [1]. Phytochemicals that are regarded as bioactive compounds in plants have been confirmed to be safe effective, relatively cheap, and recently predicted as a suitable substitute to antibiotics [2].

About 80% of the world’s inhabitants [3] and more than 90% of those listed in developing countries adopted herbal medicine for preliminary health care. Recently, medicinal plants have played an important role in pharmacological research and drug apperception [4].

Seeds from plants are potential reservoirs for secondary metabolites (bioactive compounds), proteins, fats, carbohydrates, and amino acids [5]. It has been established that the essential oils derived from the seeds of medicinal plants are abundant in phytochemicals (flavonoids, tannins, phenols, saponins, terpenoids, alkaloids, there are also generally regarded as safe and effective [6].

Gas chromatography – Mass spectrometry is an important technique that has been adapted to evaluate different phytoconstituents present in various plant extracts with their structures. This technique has superior separation potency that leads to produce a high accuracy and precision of chemical fingerprint. Moreover, quantitative data along with the coupled mass spectral database can be given by GC-MS that is of tremendous value for achieving the correlation between bioactive compounds and their applications in pharmacology [7].

*C. colocynthis*, a member of the Cucurbitaceae family, is a significant medical plant known as as “Handal” locally (in Yemen) [8].

It grows abundantly in various parts of the world (Arabian countries, Peninsula, India, Africa) [9] and has been traditionally used to treat wound numerous ailments, including wound healing [10], constipation, diabetes, oedema, fever, jaundice leukaemia, bacterial infections, cancer, and used as an abortifacient [11,12]. The seeds of *C. colocynthis* are around 6 mm in size, compressed, and smooth. They are situated in the parietal placenta. The color of seeds is light yellowish-orange to dark brown [13].

Previous literature studies have identified the presence of coumarins, tannins, terpenoids and flavonoids in the whole plant extract of *C. colocynthis* [7,14]. The current study was planned for detailed analysis of phytoconstituents in the dichloromethanolic seeds oil extract of *C. colocynthis* using GC-MS to obtained the new phytoconstituents and their pharmacological actions for understanding their medicinal properties.

## 2 Material and methods

### 2.1 Plant material

#### 2.1.1 Collection of plant material

Fresh fruits of *C colocynthis* were collected from khawlan, Sana’a city, Yemen during september 2021. *C. colocynthis* plants were identified by Dr. Ebrahim Hasn; plant taxonomist, at Sana'a University, Yemen with voucher specimen number -194-. Fruits were washed with deionized water then dried in shade.

#### 2.1.2 Sample preparation

The fruits of *C. colocynthis* were dried in shade at room temperature and then separated from the seeds from the pulp after that grinded to obtain the coarsest powder. 100 g of powdered seeds (1:5 wt of powder/Volume of solvent) were used for extraction in a Soxhlet apparatus with analytical grade refluxing solvent dichloromethane for 6 h in a water bath. Yellow seed oil extract yield 0.1% v/w base on dry weight of sample for *C. colocynthis* with viscosity at 30 °C: 29.52 mm^2^/s was stored at low temperatures for further analysis.

### 2.2 Gas chromatography – mass spectrometry analysis (GC-MS)

Separation and identification were performed on a GC-MS. The GC-MS analysis which performed using a Thermo Scientific, Trace GC Ultra/ISQ Single Quadruple MS, TG-5MS fused silica capillary column (30 m, 0.251 mm, 0.1 mm film thickness). For GC-MS detection, an electron ionization system with an ionization energy of 70 eV was used, and Helium gas was used as the carrier gas at a constant flow rate of 1 mL/min. An injection volume of 1 μL of sample is considered in the analysis. The injector and MS transfer line temperature was set at 280 °C. The oven temperature was programmed at an initial temperature of 40 °C (hold for 3 min) to 280 °C as a final temperature at an increasing rate of 5 °C/min (hold 5 min). The quantification of all the identified components was investigated using a percent relative peak area. A tentative identification of the compounds was performed based on the comparison of their relative retention time and mass spectra with those of the NIST and Wiley library data of the GC-MS system [15].

## 3 Result

### 3.1 Gas chromatography mass spectrophotometer (GC-MS) composition of *C. colocynthis* seed oil extract

**Fig. 1:**
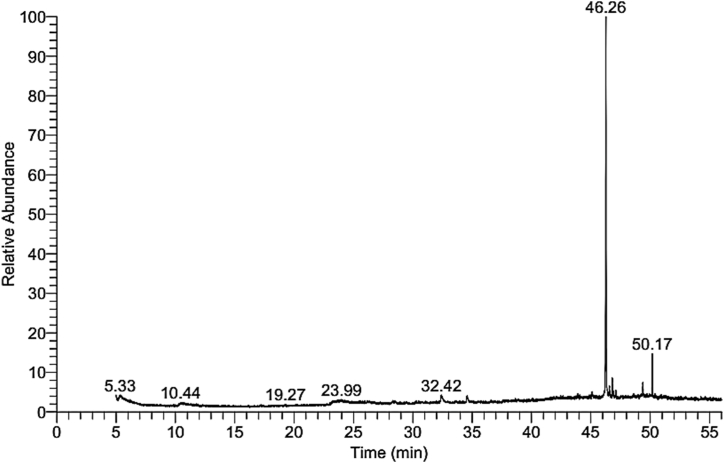
GC-MS chromatogram of dichloromethanolic seeds oil extract of *C. colocynthis* (L.).

Gas chromatography-mass spectrophotometer (GC-MS) composition of *C. colocynthis* seed oil extract. In the present study, dichloromethane seeds oil extract of *C. colocynthis* was used for GC-MS analysis. The GC-MS result of *C. colocynthis* seed oil disclosed about fifty five bioactive compounds. These compounds were submitted with their molecular formulae, molecular weight, their composition (%), and retention time determined from their peak areas (Fig. 1). Seed oil extract has shown the presence of the major twenty-four different bioactive compounds. The spectra of these bioactive compounds were matched with WEILY and NIST library’s software of GC-MS.

Diisooctylphthalate compound was found to be in the highest concentration (58.8%) followed by 2,6,10,14,18,22-Tetracosa hexaene, 2,6,10,15,19,23-hexamethyl (6.4%), 2,3-Diphenylcyclopropyl) methyl phenyl sulfoxide, trans (3.48%), Androsterone (3.09%), 1,2-Benzene dicarboxylic acid, bis(2-ethyl hexyl) ester (2.64%), Thiocarbamic acid, N,N-dimethyl, S-1,3-diphenyl-2-butenyl ester (1.8%), (4,4-Diphenyl-butyl)-(3phenyl-piperidin-4-yl)–amine (1.61%), Di-2benzothiazo Le Disulfane (1.33%), *trans*-2-phenyl-1,3dioxolane-4-methyloctadec- 9,12,15trienoate (1.3%), 2-Aza - 4,5,9,10-tetrahyd ropyrene (1.04%), and other compounds were found in trace amount (Table 1).

**Table 1:** Major bioactive compounds were identified in the dichloromethane seeds oil extract of *C. colocynthis* (L) seeds oil extracts.

No**.**	Phytochemical compound	RT (min)	M.F	M.Wt	Peak area%	Chemical structure	Library
1	*trans*-2-phenyl-1,3dioxolane-4-methyloctadec 9,12,15trienoate	5.33	C_28_H_40_O_4_	440	1.30		Wiley9
2	9-Octadecenoic acid, (2-phenyl-1,3-dioxolan-4-yl)methyl ester	5.42	C_28_H_44_O_4_	444	0.62		Wiley9
3	Penitrem A	9.98	C_37_H_44_ClNO_6_	633	0.32		Wiley9
4	1-(4-amino 1,2,5 -ox adiazol-3yl) 5-(1 piperidinylmethyl)-1h-1,2,3-triazole-4 carboxamide	12.64	C_11_H_16_N_8_O_2_	292	0.32		Wiley9
5	Zeaxanthin	24.26	C_40_H_56_O_2_	568	0.37		Wiley9
6	Lucenin 2	26.37	C_27_H_30_O_16_	610	0.38		Wiley9
7	Fenretinide	26.74	C_26_H_33_NO_2_	391	0.39		mainlib
8	Androsterone	32.41	C_19_H_30_O_2_	290	3.09		Wiley9
9	Tetraneurin F	33.67	C_19_H_26_O_7_	366	0.32		Wiley9
10	(4,4-Diphenyl-butyl)-(3phenyl-piperidin-4-yl)–amine	34.58	C_27_H3_2_N_2_	384	1.61		mainlib
11	Ceanothine C	42.10	C_26_H_38_N_4_O_4_	470	0.49		Wiley9
12	Di-2benzothiazo Le Disulfane	43.91	C_14_H_8_N_2_S_4_	332	1.33		Wiley9
13	Phorbol	44.63	C_20_H_28_O_6_	364	0.3		Wiley9
14	Lycoxanthin	45.23	C_40_H_56_O	552	0.32		Wiley9
15	Isochiapin B	45.52	C_19_H_22_O_6_	346	0.44		Wiley9
16	Diisooctyl phthalate	46.26	C_24_H_38_O_4_	390	58.53		Wiley9
17	Thiocarbamic acid, N,N-dimethyl, S-1,3-diphenyl-2-butenyl ester	46.57	C_19_H_21_NOS	311	1.83		Wiley9
18	(2,3-Diphenylcyclopro pyl)methyl phenyl sulfoxide, trans	46.80	C_22_H_20_OS	332	3.48		mainlib
19	2-Aza-4,5,9,10-tetrahyd ropyrene	46.90	C_15_H_13_N	207	1.04		Wiley9
20	Fucoxanthin	47.92	C_42_H_58_O_6_	658	0.32		mainlib
21	Phytofluene	48.97	C_40_H_62_	542	0.43		Wiley9
22	1,2 Benzene dicarboxylic acid, bis(2-ethyl hexyl) ester	49.38	C_24_H_38_O_4_	390	2.64		Wiley9
23	2,6,10,14,18,22-Tetracosa hexaene, 2,6,10,15,19,23-hexamethyl	50.17	C_30_H_50_	410	6.40		Wiley9
24	Rhodoxanthin	50.93	C_40_H_50_O_2_	562	0.51		Wiley9

RT: Retention time, M.F: Molecular formula, M.WT: Molecular weight.

### 3.2 Nature and the biological activities of some compounds from *C. colocynthis* seeds oil extract

The presence of different bioactive compounds in the dichloromethanolic seed oil extract of *C. colocynthis* justifies its types, hit spectrum, and bioactivity as in previous studies (Table 2).

**Table 2:** Nature and the biological activities of compounds from *C. colocynthis* methanolic seeds oil extract.

No.	Compound	Type of compounds	Hit spectrum	Bioactivity	Reference
1	Tetra acetyl-d-xylonic nitrile	nitriles		Antitumor and antioxidants	[16]
2	Androsterone	steroids		Enhancer for athletic performance, build body muscles, reduce fats, increase energy, maintain healthy RBCs, and increase sexual performance	[17,18]
3	diisooctyl-phthalate	Ester (phthalates**)**		Natural anticancer agents derived from plants antimicrobial activity inhibiting melanogenesis	[[19], [20], [21], [22]]
4	Fucoxanthin	carotines		Antioxidant	[[23], [24], [25]]
				Cytoproductive	
				Anti-inflammatory	
				Anticancer	
				Antiobesty	
				Antidiabetic	
				Skin productive	
				Neuropotective	
5	Isochiapin B	carotines		Antimicrobial,	[26]
				Antioxidants	
				Anticancer	
6	Phytofluene	carotenes		cancer prevention	[27]
				anti tumer	
				anti-inflammatory	
7	Zeaxanthin	carotenes		*Antioxidant*	[28]
				Inflammatory	
				Skin production	
8	Ethyl iso-allocholate	ester		Antimicrobial, anti-inflammatory	[16]
9	Ceanothine C	alkaloids			[29]

m = mass, z = charge number.

## 4 Discussion

*C. colocynthis*, a medicinal plant found in Yemen, is a rich source of various bioactive compounds. In the current study, GC. MS was used to identify the chemical constituents presents in the *C. colocynthis*. Carotenoids have been reported to prossess various biological and medicinal effects, including photoprotective, anti-angiogenic, anti-cancer, anti-diabetic, antioxidant, and anti-inflammatory properties [30]. Fucoxanthin is a pigment that accounts for about 10% of all carotenoids in nature [31]. The Yemeni *C. colocynthis* seed oil preparation contains fucoxanthin for the first time. Fucoxanthin has recently been demonstrated to have several bioactive effects, including a protective impact against oxidative stress. It was demonstrated that fucoxanthin had a protective effect against UV-B radiation and DNA damaging factors. It also exhibited anti-obesity and anti-diabetic properties, lowering blood glucose levels, reducing insulin resistance, and body weight as well as improving lipid homeostasis and having positive impact on the cardiovascular system, which was seen in the diminution of inflammatory processes, blood pressure, and levels of cholesterol and triacylglycerol [24].

The colorless carotenoid phytofluene has a less rigid conformation than other bioavailable carotenoids, which affects its sensitivity. According to Paula Mapelli-Brahm and Antonio J., popular foods like some citrus contain phytofluene [31].

Dioctylphthalates was identified by GC-MS. The spectra of the compounds were matched with NIST and Willey library. There are a few types of research on the occurrence of dioctylphthalates in plants [20]. For the first time, the GC-MS analysis of dioctylphthalate from Yemeni *C. colocynthis* is documented here for the Cucurbitaceae family. Chemicals known as dioctylphthalates have been blamed for environmental pollution. This notion, however, has progressively changed in light of the mounting evidence that phthalate compounds are unquestionably found in secondary metabolites of organisms, including plants, animals, and microorganisms [22]. Because of dioctylphthalates from plant source has low toxicity, medical effectiveness, and many natural anti-tumor agents derived from various medicinal plants, Dioctylphthalates is a growing trend in the use of medicinal plants [32,33]. Dioctylphthalates have been proven to have antibacterial and antifouling characteristics [34], and a tyrosinase inhibitor that can suppress melanogenesis [33]. As a consequence, the seed oil of *C. colocynthis* from Yemen has been regarded as a rich natural source of dioctylphthalates.

Zeaxanthin, another carotenoid identfied in the seed oil of *C. colocynthis*, has demonstrated several health benefits, antioxidant activities, and reduce inflammation [28].

Ceanothine, another carotene found in *C. colocynthis* that has a long history in folklore traditional medicine and has been used to treat a various conditions, including pressure, blood clotting, spleen pain, and cancer [29]. Overall, the presence of various bioactive compounds in the Yemeni *C. colocynthis* seed oil extract suggests its potential medicinal applications.

## 5 Conclusion

The Yemeni *C. colocynthis* (Handal) plant is a valuable source of natural compounds that have the potential to be utilized in a variety of herbal formulations, including analgesics, antipyretics, analgesics, cardiac tonics, and antiasthmatics. This study identified the formula and structure of fifty-five compounds presence in the oil extract from *C. colocynthis* seeds with twenty-four major biomolecules identified in dichloromethane seed oil extract of *C. colocynthis*. The major component identified in the extract was isooctylphthalate (58%), which exhibited strong antimicrobial effects. Therefore, *C. colocynthis* is considered to be natural source of isooctylphthalate. These finding suggest that further screening of these compounds for their pharmacological properties may be warranted, with the potential for the development new drug.

## Author contribution statement

Faten Hameed Thamer: Conceived and designed the experiments; Performed the experiments; Analyzed and interpreted the data; Contributed reagents, materials, analysis tools or data; Wrote the paper.

Noah Thamer: Analyzed and interpreted the data.

## Data availability statement

Data included in article/supplementary material/referenced in article.

## Declaration of competing interest

The authors declare that they have no known competing financial interests or personal relationships that could have appeared to influence the work reported in this paper.

## Appendix A Supplementary data

suppelimentry files for Helyion suppelimentry files for Helyion
