# Supplementary material for: Gas chromatography – Mass spectrometry (GC-MS) profiling reveals newly described bioactive compounds in Citrullus colocynthis (L.) seeds oil extracts
Source: Heliyon. 2023 Jun 3;9(6):e16861. doi: 10.1016/j.heliyon.2023.e16861 (PMC10360964; doi:10.1016/j.heliyon.2023.e16861)
Supplement: suppelimentry files for Helyion [file mmc1.pdf]

Table S**1.** All bioactive compounds identified of dichloromethane *Citrullus colocynthis (L)* seeds oil

**No.**	Phytochemical compound	RT (min)	M.F	M.Wt	Peak area%	Chemical structure	**Library**
1	trans-2-phenyl-1,3dioxolane-4-methyloctadec 9,12,15trienoate	5.33	$C_{28}H_{40}O_4$	440	1.30	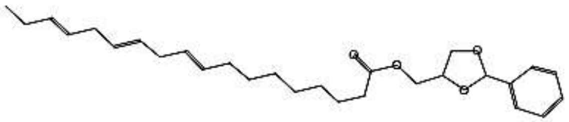	Wiley9
2	9-Octadecenoic acid,(2-phenyl-1,3-dioxolan-4-yl)methyl ester	5.42	$C_{28}H_{44}O_4$	444	0.62	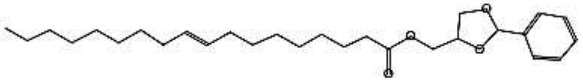	Wiley9
3	Penitrem A	9.98	$C_{37}H_{44}ClNO_6$	633	0.32	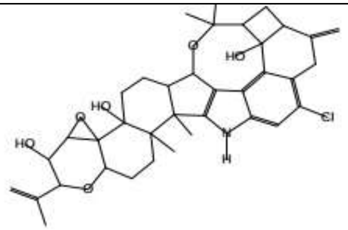	Wiley9
4	1-(4-amino 1,2,5 -ox adiazol-3yl) 5-(1 piperidinylmethyl)-1h-1,2,3-triazole-4 carboxamide	12.64	$C_{11}H_{16}N_8O_2$	292	0.32	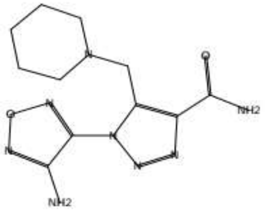	Wiley9

5	B-N-acetylneuraminic me ester-2-ME-8,9 meboronate-3,7-di tms	21.48	$C_{20}H_{40}BNO_9Si_2$	505	0.33	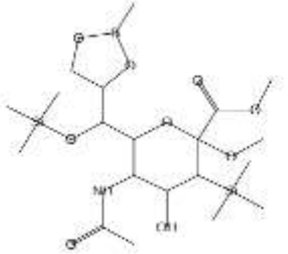	Wiley9
6	Dodecachloro-3,4-benzo phenanthrene	23.93	$C_{18}Cl_{12}$	636	0.31	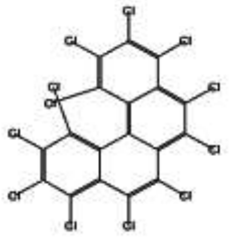	Wiley9
7	Zeaxanthin	24.26	$C_{40}H_{56}O_2$	568	0.37	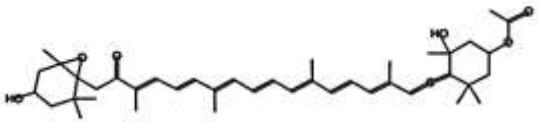	Wiley9
8	Lucenin 2	26.37	$C_{27}H_{30}O_{16}$	610	0.38	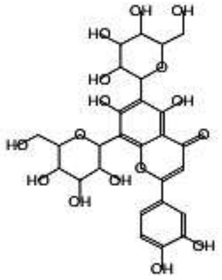	Wiley9

9	Fenretinide	26.74	$C_{26}H_{33}NO_2$	391	0.39	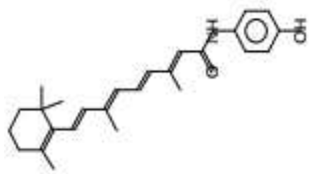	mainlib
10	2,4,7-Trinitro-9-fluoren one 2,4 dinitrophenylhydra zone	28.31	$C_{19}H_9N_7O_{10}$	495	0.5	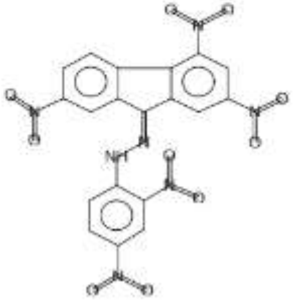	mainlib
11	Eicosane, 2-cyclohexyl-	28.5	$C_{26}H_{52}$	364	0.41	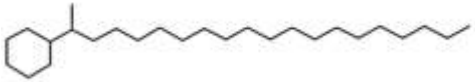	mainlib
12	N,N,N'-TRIMETHYL-N '-(4-METHOXY-CIS-C INNAMOYL-PUTRES CIN 8	29.5	$C_{17}H_{26}N_2O_2$	290	0.3	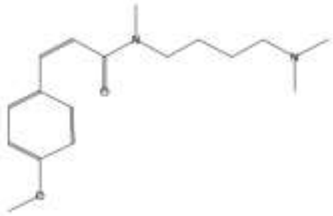	Wiley9

13	Chloro-(3-chloro-4,5-di hydro-isoxazol-5-yl)-acetic acid, ethyl ester	31.09	$C_7H_9Cl_2NO_3$	225	0.32	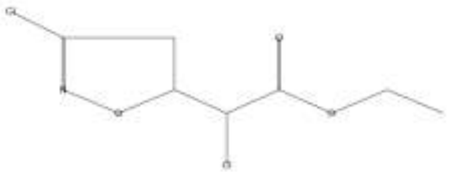	Wiley9
14	Androsterone	32.41	$C_{19}H_{30}O_2$	290	3.09	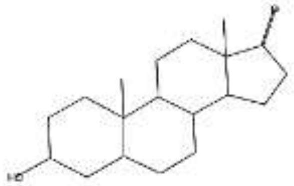	Wiley9
15	5H-Cyclopropa[3,4]benz[1,2-e]azulen-5-one, 1,1a,1b,4,4a,7a,7b,8,9,9a-decahydro-7b,9,9a-t rihydroxy-3-(hydroxymethyl)-1,1,6,8-tetramethyl-,[1aR-(1aà,1bá,4aà,7aà,7 bà,8à,9á,9aà)]-	33.58	$C_{20}H_{28}O_5$	348	0.54	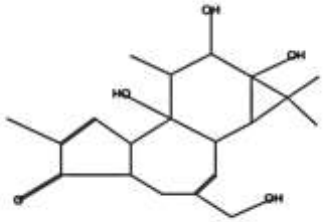	mainlib
16	Tetraneurin F	33.67	$C_{19}H_{26}O_7$	366	0.32	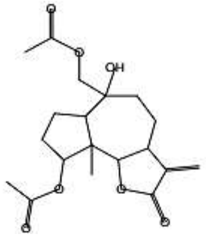	Wiley9

17	N-D2-METHYL-10,10,22,22,-D4-Dihydrovindolininol	34.06	$C_{21}H_{22}D_6N_2O$	324	0.32	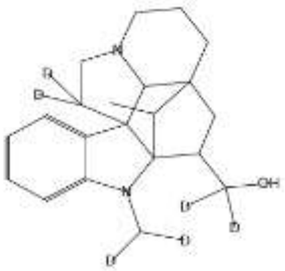	Wiley9
18	(4,4-Diphenyl-butyl)-(3phenyl-piperidin-4-yl) –amine	34.58	$C_{27}H3_2N_2$	384	1.61	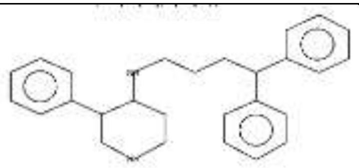	mainlib
19	2-[(Benzo[1,3]dioxole-4-carbonyl)-amino]-3-hydroxy-propionic acid	36.58	$C_{11}H_{11}NO_6$	253	0.36	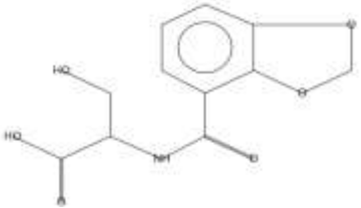	Wiley9
20	Androstan-17-one,3-hydroxy-,(3à,5á)-(CAS)	38.84	$C_{19}H_{30}O_2$	290	0.32	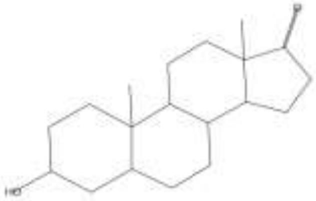	Wiley9

21	4-O-Methyl-12b,13,20-triacetoxy-2,9-dihydroxy-3a-carboxy-2,3-secotigla-1(10),6-diene-3,9-lactone	40.07	$C_{27}H_{36}O_{10}$	520	0.45	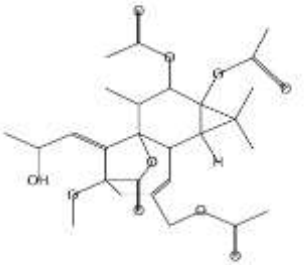	mainlib
22	Ceanothine C	42.10	$C_{26}H_{38}N_4O_4$	470	0.49	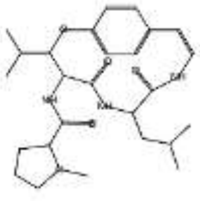	Wiley9
23	2-[3,4-Bis(dodecyloxy)phenyl]-4,4,5,5-tetramethyl-1,3,2-dioxaborolane	43.7	$C_{36}H_{65}BO_4$	572	0.94	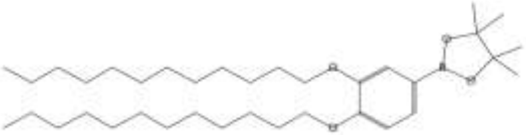	Wiley9
24	Di-2benzothiazo Le Disulfane	43.91	$C_{14}H_8N_2S_4$	332	1.33	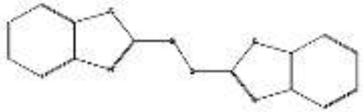	Wiley9
25	Phorbol	44.63	$C_{20}H_{28}O_6$	364	0.3	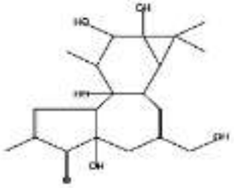	Wiley9

26	4-Carbomethoxy-4-[2,2carbomethoxyvinyl) benzyl]-3-methoxy-2,5-cyclohexadien-1-one	45.11	$C_{20}H_{20}O_6$	356	1.67	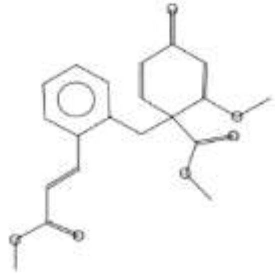	mainlib
27	Lycoxanthin	45.23	$C_{40}H_{56}O$	552	0.32	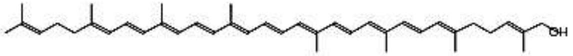	Wiley9
28	Isochiapin B	45.52	$C_{19}H_{22}O_6$	346	0.44	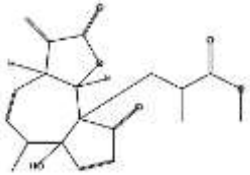	Wiley9
29	Diisooctyl phthalate	46.26	$C_{24}H_{38}O_4$	390	58.53	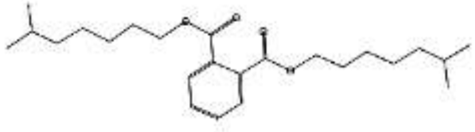	Wiley9
30	Thiocarbamic acid, N,N-dimethyl, S-1,3-diphenyl-2-butenyl ester	46.57	$C_{19}H_{21}NOS$	311	1.83	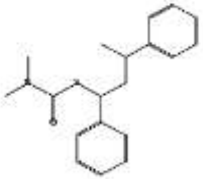	Wiley9

31	(2,3-Diphenylcyclopropyl)methyl phenyl sulfoxide, trans	46.80	$C_{22}H_{20}OS$	332	3.48	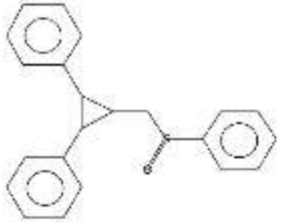	mainlib
32	2-Aza-4,5,9,10-tetrahyd ropyrene	46.92	$C_{15}H_{13}N$	207	1.04	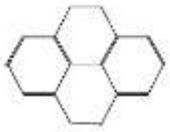	Wiley9
33	Benzene,1,1'-[3-(2-phenylethylidene)-1,5-pentanediyl]bis-	47.10	$C_{25}H_{26}$	326	1.22	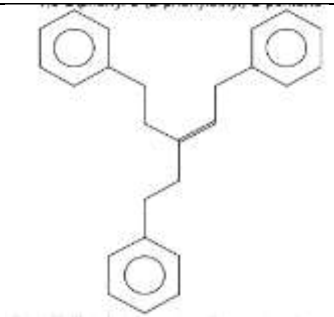	mainlib
34	Fucoxanthin	47.92	$C_{42}H_{58}O_6$	658	0.32	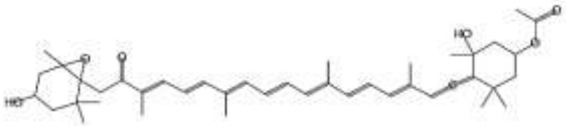	mainlib

35	1H-Cyclopropa[3,4]benz[1,2-e]azulene-5,7b, 9,9a-tetrol,1a,1b,4,4a,5,7a,8,9-octahydro-3-(hydroxymethyl)-1,1,6,8-tetramethyl-,5,9,9a-triacetate,[1aR-(1aà,1bá,4aá,5á,7aà,7bà,8à,9á,9aà)]-	48.62	$C_{26}H_{36}O_8$	476	0.73	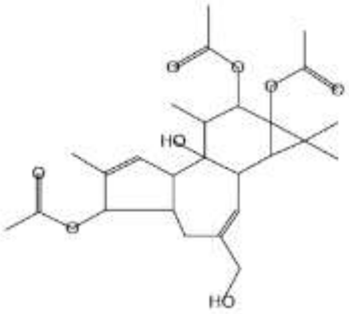	mainlib
36	2-CHLOROSANTONIN	48.73	$C_{15}H_{17}ClO_3$	280	0.37	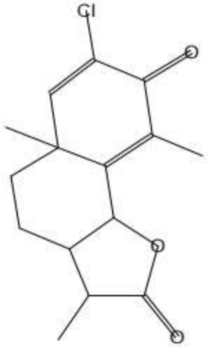	Wiley9
37	Phytofluene	48.97	$C_{40}H_{62}$	542	0.43	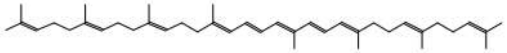	Wiley9

38	1H-Cyclopropa[3,4]benz[1,2-e]azulene-4a,5, 7b,9,9a(1aH)-pentol,3-[(acetyloxy)methyl]-1 b,4,5,7a,8,9-hexahydro-1,1,6,8-tetramethyl-, 5,9,9a-triacetate,[1aR-(1aà,1bá,4aá,5á,7 aà,7bà,8à,9á,9aà)]-	49.23	$C_{28}H_{38}O_{10}$	534	0.54	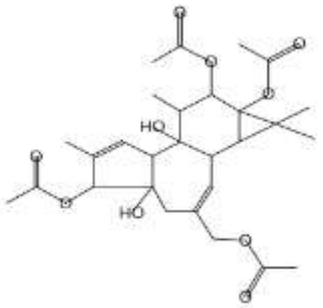	mainlib
39	1,2 Benzene dicarboxylic acid, bis(2-ethyl hexyl) ester	49.38	$C_{24}H_{38}O_4$	390	2.64	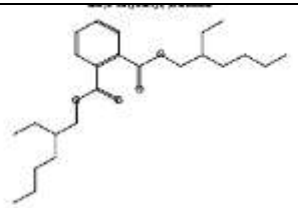	Wiley9
40	Lucenin 2	49.72	$C_{27}H_{30}O_{16}$	610	0.41	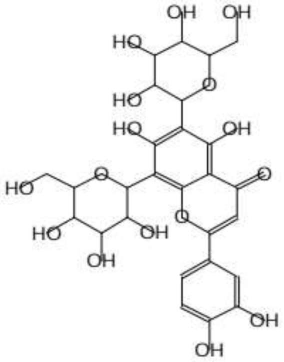	Wiley9

41	2,6,10,14,18,22-Tetracosa hexaene, 2,6,10,15,19,23-hexamethyl	50.17	$C_{30}H_{50}$	410	6.40	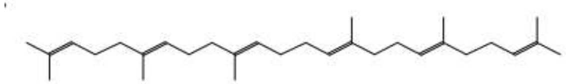	Wiley9
42	17-(1,5-Dimethylhexyl)-10,13-dimethyl-3-styrylhexadecahydrocyclopenta[a] phenanthren-2-one	50.39	$C_{35}H_{52}O$	488	0.7	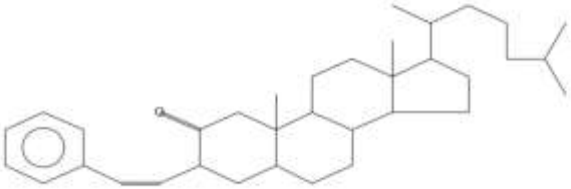	mainlib
43	à-D-Galactopyranoside,methyl2,3-bis-O-(trimethylsilyl)-, cyclicbutylboronate (CAS)	50.82	$C_{17}H_{37}BO_6Si_2$	404	0.35	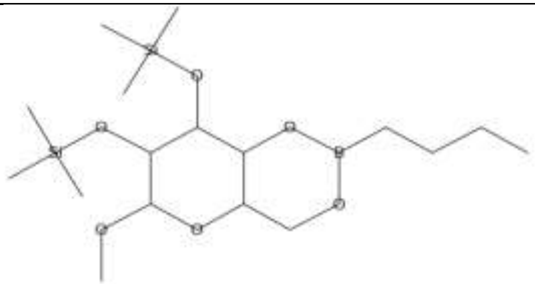	Wiley9
44	Rhodoxanthin	50.93	$C_{40}H_{50}O_2$	562	0.51	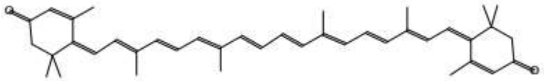	Wiley9
45	D-Glucose,6-O-à-D-galactopyranos yl-,bis-O-(trimethylsilyl)deriv.,cyclic tris(methylboronate)(CAS)	50.96	$C_{21}H_{41}B_3O_{11}Si_2$	558	0.51	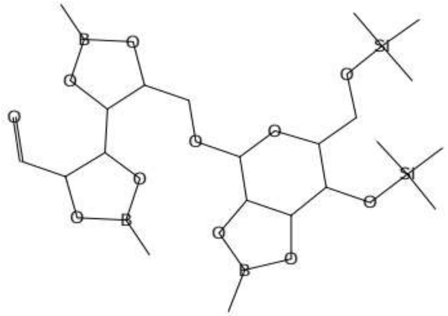	Wiley9

46	(22R)-6á,11á,21-Trihydroxy-16à,17à-propylmethylenedioxypregna-1,4-diene-3,20-dione	50.98	$C_{25}H_{34}O_7$	446	0.7	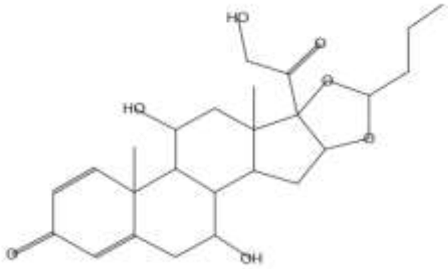	mainlib
47	2,6,10,14,18,22-Tetracosahexaene, 2,6,10,15,19,23-hexamethyl-, (all-E)- (CAS)	51.10	$C_{30}H_{50}$	410	6.10	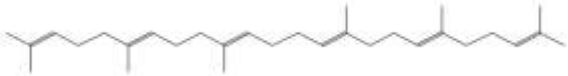	Wiley9
48	7,8-Epoxylanostan-11-ol, 3-acetoxy-	51.18	$C_{32}H_{54}O_4$	502	0.41	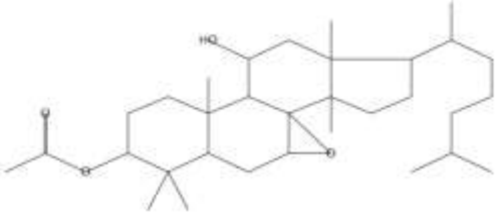	mainlib
47	Rhodopin	51.33	$C_{40}H_{58}O$	554	0.41	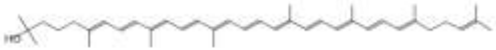	mainlib

48	1H-Cyclopropa[3,4]benz[1,2-e]azulene-5,7b, 9,9a-tetrol,1a,1b,4,4a,5,7a,8,9-octahydro-3-(hydroxymethyl)-1,1,6,8-tetramethyl-,5,9,9a-triacetate,[1aR-(1aà,1bá,4aá,5á,7aà,7bà,8à,9á,9aà)]-	51.59	$C_{26}H_{36}O_8$	476	0.43	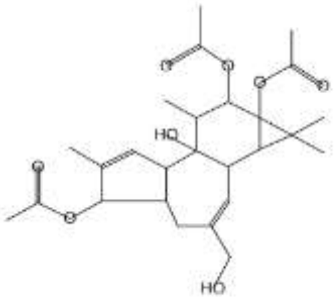	mainlib
49	(-)-Corlumine	52.53	$C_{21}H_{21}NO_6$	383	0.43	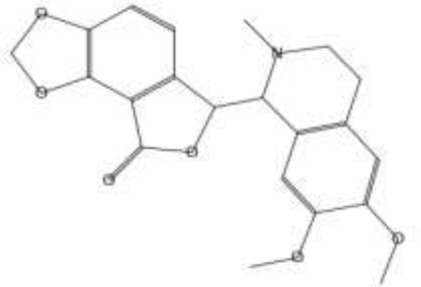	mainlib
50	8,14-Seco-3,19-epoxyandrostane-8,14-dione, 17-acetoxy-3á-methoxy-4,4-dimethyl-	52.8	$C_{24}H_{36}O_6$	420	0.43	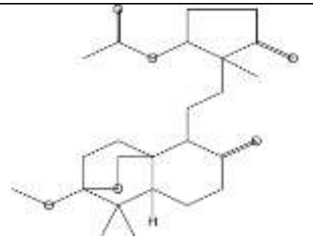	Wiley9

51	Aspidospermidin-21-ol,1-acetyl-15,16,17-trimethoxy-, acetate (ester)(CAS)	53.98	$C_{26}H_{36}N_2O_6$	472	0.4	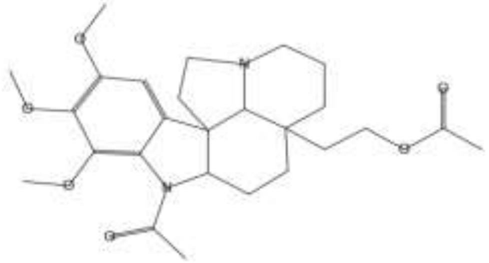	Wiley9
52	2-(3-Acetoxy-4,4,10,13,14-pentamethyl-2,3,4,5,6,7,10,11,12,13,14,15,16,17-tetradecahydro-1H-cyclopenta[a]phenanthren-17-yl)-propioni	54.1	$C_{27}H_{42}O_4$	430	0.4	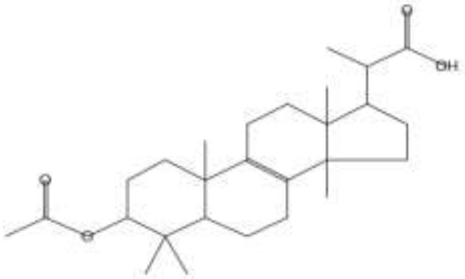	Wiley9
53	3-Acetoy-8-deacetoxy-yunaconitine	54.24	$C_{35}H_{49}NO_{10}$	643	0.30	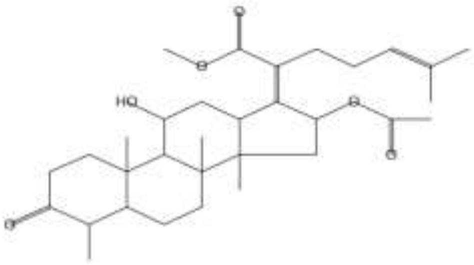	Wiley9

54	2-(16-Acetoxy-11-hydroxy-4,8,10,14-tetramethyl-3-oxohexadecahydrocyclopenta[a]phenanthren-17-ylidene)-6-methyl-hept-5-enoic acid,methyl ester	54.50	$C_{32}H_{48}O_6$	528	0.3	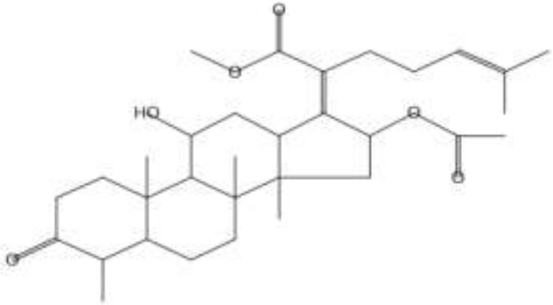	Wiley9
55	N'-(2-HYDROXY-5-METHOXYBENZYLIDENE)-2-(4-CHLOROPHENOXY)ACETHYDR AZIDE	54.62	$C_{16}H_{15}ClN_2O_4$	334	0.69	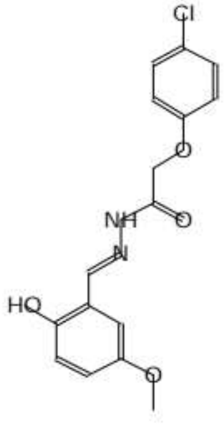	
